# On the Use of the Ergot of Rye in Uterine Leucorrhœa

**Published:** 1829-05

**Authors:** Marshall Hall


### THE LONDON
### Medical and Physical Journal.

No. 363, VOL. LXI.] MAY, 1829. [No. 35, *New Series*.

For many fortunate discoveries in medicine, and for the detection of numerous errors, the world is indebted to the rapid circulation of Monthly Journals; and there never existed any work, to which the Faculty, in Europe and America, were under deeper obligations than to the *Medical and Physical Journal of London*, now forming a long, but an invaluable series.—RUSH.

### ORIGINAL PAPERS, AND CASES,

### OBTAINED FROM PUBLIC INSTITUTIONS AND OTHER AUTHENTIC SOURCES.

### UTERINE LEUCORRHŒA.

*On the Use of the Ergot of Rye in Uterine Leucorrhœa.* By MARSHALL HALL, M.D. F.R.S.E. &c. (In a Letter to JOHN NORTH, Esq.)

THE bearer of this communication is the patient whom I promised to send to you, and whose case was promptly relieved by the ergot of rye. She will herself describe the truly deplorable state in which she had long been, before she began the use of this remedy; but I believe the following particulars, which I transcribe from my notes, will be found accurately to coincide with the account she will give you.

From August 1824, after a protracted labour, to September, she became subject to profuse menorrhagic discharges, during which large coagula of blood were continually expelled; and after which there was the most profuse leucorrhœa. She became, of course, as blanched, thin, and feeble as a young person could be expected to be from such excessive drains upon the vascular system. The leucorrhœa only ceased, to yield to the hemorrhagy, and the latter gradually to pass into leucorrhœa; so that the patient could never be without the usual bandage for the reception of uterine or vaginal discharges: sometimes the case ceased to be menorrhagic, but only because the hemorrhagy itself was protracted for many weeks, once four, once six, without intermission, from March 1827, to September 1828, however, the periods were quite regular.

In September 1828, this patient began to use a cold lotion, applied over the uterine region. The next catamenial period occurred a few days afterwards; it was attended by excessive hemorrhage for twelve days, a faintly tinged discharge for three days more, and then by profuse leucorrhœa.

At the latter end of October, five grains of the ergot of rye were prescribed to be taken three times a day, in pills, beginning after the catamenia had flowed three days. Little effect was observed. The medicine was increased to four times a day, at the *beginning* of the ensuing catamenial period. The discharge was evidently checked. The ergot being continued, it greatly abated the leucorrhœa.

The ergot having been omitted, was resumed at the commencement of the next expected catamenial period. The flow was retarded in its appearance for four days, was altogether moderate in its quantity, free from coagula, unfollowed by the faintly tinged discharge, or by leucorrhœa.

This was observed in December. Three months have elapsed since that period. The patient has been free from all menorrhagia, and all undue flow of the catamenia, and from leucorrhœa. The medicine has been regulated by herself, being omitted and resumed at intervals. The colour, the strength, and the flesh are restored, and the symptoms so characteristic of vascular exhaustion have gradually, but totally subsided.

I have not since had occasion to try the effect of the ergot in menorrhagia; but I have prescribed it in many cases of leucorrhœa; in all, with the most prompt and decided advantage. The benefit which accrues from the ergot is indeed frequently experienced, in the most marked manner, in the space of five days; and I have generally recommended this medicine to be taken for a somewhat longer period than this, then to be abandoned for a few days, and again resumed.

In the first case of leucorrhœa in which I gave it, the patient had suffered for several years from returns of this affection, and, for three weeks previously to her visit to me, it had subsisted in such a degree as to incapacitate her for her occupation as a servant: she had become pale and weak, and affected with sad headach. After an aperient, this patient took five grains of the ergot four times a day: she was better in three days, much better after the lapse of a week, and perfectly well at the end of a fortnight. Nothing could be more marked than the prompt efficacy of the remedy.

The other cases it is quite unnecessary to detail.

It will, of course, be necessary to give the ergot with discrimination. We could expect no good from it in cases of an inflammatory or organic nature, or in vaginal discharges not uterine. It is not *every* sanguineous or white discharge which can be expected to be remedied by the ergot. The former should, I think, be distinctly catamenial or menorrhagic, or at least independent of inflammation or organic disease; the latter uterine, and not merely vaginal, and, of course, not dependent on any continued cause, as undue lactation. In such cases, well marked and distinguished, I believe the ergot of rye will be found to be a most useful remedy.

It is quite plain, contrary to the opinion of a late writer on the virtues of the ergot of rye, that this substance exerts its power over the state of the uterus in other circumstances besides that of approaching contraction. A state of what may be deemed undue relaxation or want of tone in this organ, seems to be under the immediate influence of the ergot.

I may here add, that I have tried this remedy in a distinct case of chlorotic amenorrhœa, without the least good effect.

15, *Keppel street, Russell square;*
*April* 11*th*, 1829.

---

MIDWIFERY.

